# Classifications for Proliferative Vitreoretinopathy (PVR): An Analysis of Their Use in Publications over the Last 15 Years

**DOI:** 10.1155/2016/7807596

**Published:** 2016-06-27

**Authors:** Salvatore Di Lauro, Mustafa R. Kadhim, David G. Charteris, J. Carlos Pastor

**Affiliations:** ^1^IOBA (Eye Institute), University of Valladolid, 47011 Valladolid, Spain; ^2^Hospital Clínico Universitario de Valladolid, 47003 Valladolid, Spain; ^3^Moorfields Eye Hospital, London EC1V 2PD, UK

## Abstract

*Purpose*. To evaluate the current and suitable use of current proliferative vitreoretinopathy (PVR) classifications in clinical publications related to treatment.* Methods*. A PubMed search was undertaken using the term “proliferative vitreoretinopathy therapy”. Outcome parameters were the reported PVR classification and PVR grades. The way the classifications were used in comparison to the original description was analyzed. Classification errors were also included. It was also noted whether classifications were used for comparison before and after pharmacological or surgical treatment.* Results*. 138 papers were included. 35 of them (25.4%) presented no classification reference or did not use any one. 103 publications (74.6%) used a standardized classification. The updated Retina Society Classification, the first Retina Society Classification, and the Silicone Study Classification were cited in 56.3%, 33.9%, and 3.8% papers, respectively. Furthermore, 3 authors (2.9%) used modified-customized classifications and 4 (3.8%) classification errors were identified. When the updated Retina Society Classification was used, only 10.4% of authors used a full C grade description. Finally, only 2 authors reported PVR grade before and after treatment.* Conclusions*. Our findings suggest that current classifications are of limited value in clinical practice due to the inconsistent and limited use and that it may be of benefit to produce a revised classification.

## 1. Introduction

Proliferative vitreoretinopathy (PVR) remains the major complication after retinal detachment surgery [1–3]. PVR was identified as an independent clinical entity in 1983 by the Retina Society Terminology Committee and a classification was created [4], based on the condition formerly named “massive vitreous traction” or “massive periretinal proliferation” [5–7]. This classification divided PVR into four stages, A, B, C, and D, apparently by increasing its severity, from minimal to massive PVR (Table 1). Nevertheless, this classification had numerous limitations. It did not consider the location of the vitreoretinal traction and the magnitude of the contraction. In addition, some of the stages provide a false idea of severity; for instance, D4 caused by a localized epiretinal membrane could be more easily treated by surgery than a C1 caused by intraretinal changes [8, 9]. In 1989, the Silicone Study Group introduced a new classification [10] including some characteristics, such as the location, anterior or posterior, and the type of contraction (Table 2). Classification was then updated in 1991 [11] according to modifications proposed by the Silicone Study Group and also by other authors [12] (Table 3). This classification appears to be difficult to use in clinical practice and may not offer any special advantage for decision-making in relation to the treatment of the disease. Moreover, the classifications might have prevented advances in the understanding of the disease pathogenesis. For instance, because the Retina Society Committee defined PVR as a “proliferative disease,” many treatments based on the inhibition of cell proliferation were developed for more than 20 years, none of which appears to have produced a significant clinical advance. Therefore, a review of both the classification and the pathogenesis of PVR appears to be appropriate to aid the development of new treatments [3].

There is a clinical impression that over the last 15 years clinicians have abandoned current PVR classifications. Thus, the purpose of this study has been to evaluate the current use of PVR classifications in papers dealing with clinical practice and therapy as an indirect measure of the degree of usefulness of existing classifications.

## 2. Methods

A search in PubMed for papers published between January 2000 and January 2014 was undertaken by two independent researchers. The basic term “proliferative vitreoretinopathy therapy” has been used. Inclusion criteria comprised human studies published in English, French, and Spanish on PVR or retinal detachment with specific mention to PVR. Prospective randomized and nonrandomized clinical studies, retrospective clinical studies, short series, and pilot studies were included. A manual search of related articles was also performed through references reported in each article. Outcome parameters were reported PVR classification, if any, and reported PVR grades. We also analyzed how the different authors interpreted the classifications, comparing the results they provided with the original description. Therefore, we investigated if they used all the characteristics (grade [A, B, C], PVR localization, extension in hours, and type) of the updated Retina Society Classification or if they were used only partially. Classification errors were also included. Furthermore, it was also noted whether classifications were used both before and after an eventual PVR pharmacological or surgical treatment. Data were extracted using a custom-made data sheet performed with agreement between authors.

## 3. Results

Preliminary search identified a total of 219 publications, 81 of which were eliminated as they did not fulfill the inclusion criteria. Thus, 138 publications were finally included and analyzed. 35 articles (25,4%) did not undergo detailed analysis because there was no reference to the type of PVR classification used by the authors or because no classification was used (generic terms such as initial or severe PVR or simply PVR were adopted, without using any grading system). 103 publications (74,6%) used standardized PVR classifications and they were analyzed in detail. The most used classification was the updated Retina Society Classification [11] (58 cites; 56,3%), followed by the first Retina Society Classification [4] (35 cites; 33,9%). 4 publications (3,8%) used the Silicone Study Classification (Table 4) [10]. In addition, 3 authors (2,9%) used modified or customized classifications [13–15]. In 4 publications (3,8%), errors in the stated classification were identified [13, 16–18]: in two papers [16, 18], the authors cite the updated Retina Society (‘91) but in fact they used the first Retina Society Classification (‘83), Koerner et al., after citing the updated Retina Society Classification used a customized one [13], and Roldán-Pallarés et al. cite the two Retina Society Classifications at the same time [17].

Among the papers using the updated Retina Society Classification, the most documented was grade C PVR (48 articles, 83%), while grades B and A were less frequently documented (15 (26%) and 7 (12%) articles, resp.). Of the publications documenting only C grade, the classification of subtypes was infrequently used. In 5 of 48 publications (10,4%), there was a full C grade description in terms of localization (anterior or posterior), extension (in clock hours), and type (focal, diffuse, subretinal, circumferential, or anterior displacement). A similar finding was observed when authors used the first Retina Society Classification (35 papers): only C and D grades were frequently reported (27 and 10 papers, resp.), while grades A and B were infrequently used (4 and 7 articles, resp.). When the Silicone Study Classification was used, only grade C was mentioned (in 2 out of 4 articles). Finally, there were 2 publications (1,9%) in which the standard classifications were used to assess changes in PVR status after any treatment, reporting PVR grade before and after it [19, 20]. The remaining authors used the classifications only to describe PVR status, rather than using them to analyze the improvement or worsening of the previous stage after the applied treatment.

## 4. Discussion

Classifications of PVR were developed to provide clinicians with a useful tool to compare results of treatments. They are, to date, purely descriptive and do not reflect the pathobiology of this complex vitreoretinal disease. The original classification of 1983 [4], which was relatively simple to use, induces errors in the estimating severity in some cases. As mentioned, D grades were based on the ophthalmoscopic appearance of the detached retina, and experience demonstrated that some of these cases could be relatively easily solved by peeling localized epiretinal membranes although, in some cases, classified as C, intraretinal severe changes prevent reattachment, unless complicated surgical techniques were used. Subsequent classifications [10, 11] were more detailed and therefore more complicated to use on a routine basis. They incorporated the anterior PVR forms, which add severity to the case, but until now no one has incorporated the intraretinal changes, observed in many surgical retinal samples [8, 9], which may prevent the anatomical reattachment of the retina and require retinectomy. Another important limitation is that they do not provide information on the activity of the process, although it has been observed that PVR progresses through several stages [1–3]. This may be crucial in estimating the risk of reproliferation after surgery or when surgeons schedule removing silicone oil, and no data on the chronology of events is included in any classification. Furthermore, current classifications are not prognostic and do not correlate with visual prognosis or anatomical success after surgical treatment.

Moreover, some of these classifications can be difficult to use in clinical practice, due to their complexity (grade, localization, extension, type, etc.) and because they may not provide useful information they have been largely abandoned by clinicians. Our findings in this study demonstrate that, in clinical research, investigators are using them inconsistently and reporting limited observations of stages (mostly grade C).

The results of this study suggest that the current classifications are of limited value. Some authors simply do not use any, preferring generic terms, such as minimal, moderate, and severe PVR [21, 22]. Furthermore, early stages of PVR (named as grades A and B), which are common to all classifications, are not used, and most authors refer only to most advanced stages, basically grade C [20, 23]. Moreover, the presence of classification errors could indicate that there is confusion concerning their use [13, 16–18]. An important relevant finding is that only a limited number of clinicians appropriately and fully use the current updated classification of 1991 [24–27], while the majority of authors avoid using the added characteristics of localization, extension, and type, limiting the description to the grade. In addition, many colleagues still use the first reported classification [4], probably because although it presents many limitations, it is easier to use compared to the last version [23, 28]. Additionally, some authors decided to use alternative classification, probably to avoid problems evaluating PVR stages [14].

The use of multiple classifications makes the efficient communication between clinicians and the comparison of different studies very problematic. As mentioned, one of the aims of this study was to evaluate the usefulness of these classifications for PVR management with surgery and other adjunctive treatments. Unfortunately, many ophthalmologists did not use the normalized classifications for comparing the outcomes using non-clearly defined terms such as PVR recurrence [29, 30].

## 5. Conclusions

Our findings showed the inconsistent and limited use of the current PVR classifications suggesting that it may be of benefit to produce a revised classification incorporating, if possible, the new knowledge on PVR which has been published since 1991, pointing out new potential targets for therapeutic agents distinct from those, mainly proliferative agents, targeted by the original description of this disease. Thus, it is possible that we could reduce its prevalence after retinal detachment surgery and to improve the anatomical and functional results of this disease which resists the attempts of both basic researchers and clinicians for more than 30 years.

## Figures and Tables

**Table 1:** Classification from the Retina Society Terminology Committee (1983) [4]. This classification subdivided PVR into four stages, A, B, C, and D, apparently increasing in severity, from minimal to massive PVR. Modified from The Retina Society Terminology Committee, [4].

Retina Society Terminology Committee (1983)	
Grade	Clinical signs
A (minimal)	Vitreous haze and pigment clumps

B (moderate)	Surface retinal wrinkling, rolled edges of the retinal, retinal stiffness, and vessel tortuosity

C (marked)	Full-thickness fixed retinal folds in
(i) C-1	(i) one quadrant,
(ii) C-2	(ii) two quadrants,
(iii) C-3	(iii) three quadrants

D (massive)	Fixed retinal folds in four quadrants that result in
(i) D-1	(i) a wide funnel shape,
(ii) D-2	(ii) a narrow funnel shape,
(iii) D-3	(iii) closed funnel without view of the optic disc

**Table 2:** Silicone Study Classification (1989). This classification scheme included proliferative membranes in the preequatorial region and on the vitreous base (anterior PVR). Attention was paid to the quantitative assessment of PVR (number of clock hours of the retina involved by membranes). Minimal (grade A) and moderate (grade B) classifications remained unchanged, but grades C and D were replaced by grades P and A (posterior and anterior forms). Moreover, grades P and A were further defined by the presence of “types” of contraction. The extension of each grade was assessed by the number of clock hours of the involved retina. Modified from [10].

Silicone Study Classification (1989)	
Grade and type	Clinical signs
A	Vitreous haze and pigment clumps

B	Surface retinal wrinkling, rolled edges of the retinal, retinal stiffness, and vessel tortuosity

P P1: 1 quadrant (1–3 clock hours) P2: 2 quadrants (4–6 clock hours) P3: 3 quadrants (7–9 clock hours) P4: 4 quadrants (10–12 clock hours) (i) Type 1 (focal) (ii) Type 2 (diffuse) (iii) Type 3 (subretinal)	Starfolds and/or diffuse Contraction in posterior retina and/or subretinal membrane in posterior retina (i) Starfold (ii) Confluent irregular retinal folds in posterior retina; remainder of retina drawn posterior; optic disc that may not be visible (iii) “Napkin ring” around disc or “clothesline” elevation of retina

A A1: 1 quadrant (1–3 clock hours) A2: 2 quadrants (4–6 clock hours) A3: 3 quadrants (7–9 clock hours) A4: 4 quadrants (10–12 clock hours) (i) Type 4 (circumferential) (ii) Type 5 (perpendicular) (iii) Type 6 (anterior)	Circumferential and/or perpendicular and/or anterior traction in anterior retina (i) Irregular retinal folds in the anterior retina; series of radial folds more posteriorly; peripheral retina within vitreous base stretched inward (ii) Smooth circumferential fold of retina at insertion of posterior hyaloids (iii) Circumferential fold of retina at insertion of posterior hyaloids pulled forward; trough of peripheral retina anteriorly; ciliary processes stretched with possible hypotony; iris retracted

Quadrants refer to the circumferential area of retina directly involved in contraction. One quadrant, 1–3 clock hours; two quadrants, 4–6 clock hours; three quadrants, 7–9 clock hours; and four quadrants, 10–12 clock hours. Clock hours need not necessarily be contiguous.

**Table 3:** The updated Retina Society Classification (1991). The revised classification incorporated changes proposed by the Silicone Study Group [10] and modifications proposed also by other authors [12]. Three grades of supposed increasing severity were described, emphasizing the posterior and anterior locations of proliferation. The new classification kept grades A and B, modified grade C, and eliminated grade D. According to the Silicone Study Classification, a more detailed description of grade C (posterior and anterior) PVR was made by adding the types of contraction, the extent of which was detailed by using clock hours instead of quadrants. Modified from [11].

The updated Retina Society Classification [11]	
Grade and type	Clinical signs
A	Vitreous haze, pigment clumps, and pigment clusters on inferior retina

B	Wrinkling of inner retinal surface, retinal stiffness, vessel tortuosity, rolled and irregular edge of retinal break, and decreased mobility of vitreous

CP (posterior) (i) Type: (a) Focal (b) Diffuse (c) Subretinal	Full-thickness retinal folds or subretinal strands posterior to equator (1–12 clock hours involvement) (i) Starfolds posterior to vitreous base (ii) Confluent starfolds posterior to vitreous base; optic disc that may not be visible (iii) Proliferation under the retina; annular strand near disc; linear strands; moth-eaten-appearing sheets

CA (anterior) (i) Type: (a) Circumferential (b) Anterior	Full-thickness retinal folds or subretinal strands anterior to equator (1–12 clock hours involvement), anterior displacement, and condensed vitreous strands (i) Retina contraction inwards at the posterior edge of the vitreous base; with central displacement of the retina; peripheral retina stretched; posterior retina in radial folds (ii) Snterior contraction on the retina at the vitreous base; ciliary body detachment and epiciliary membrane; iris retraction

**Table 4:** Distribution of the used classifications for each year in papers published between January 2000 and January 2014. No data are shown about 2014 because there were no papers published at this time.

Year of publication	Retina Society Classification (1983)	Silicone Study Classification (1989)	Updated Retina Society Classification (1991)
2000	4	1	0
2001	1	0	3
2002	6	0	2
2003	2	0	1
2004	1	0	6
2005	5	0	4
2006	4	0	1
2007	1	1	7
2008	3	1	10
2009	0	0	6
2010	3	0	10
2011	2	0	2
2012	1	0	0
2013	2	1	6

	Tot.: 35	Tot.: 4	Tot.: 58
